# Systemic sclerosis in a patient with pityriasis rubra pilaris

**DOI:** 10.4314/pamj.v6i1.69070

**Published:** 2010-08-09

**Authors:** Frikha Faten, Frigui Makram, Masmoudi Hatem, Turki Hamida, Bahloul Zouhir

**Affiliations:** 1 Department of Internal Medicine, Hospital of Hedi Chaker, 3029 Sfax, Tunisia; 2 Laboratory of Immunology, Hospital of Habib Bourguiba 3029 Sfax,Tunisia; 3 Department Of Dermatology, Hospital of Hedi Chaker, 3029 Sfax, Tunisia

**Keywords:** Systemic sclerosis,, pityriasis rubra pilaris,, skin disease,, autoimmune disease,, acroosteolysis,, rare diseases

## Abstract

Pityriasis rubra pilaris (PRP) is a rare, chronic erythematous squamous disorder of unknown etiology. It has been found in association with several autoimmune diseases, including thyroiditis, myositis, myasthenia gravis and vitiligo. Herein we report a case of systemic sclerosis in a patient with classic adult pityriasis rubra pilaris. A 38 year old woman with classic adult type 1 pityriasis rubra pilaris (PRP) developed progressive skin thickening of the trunk, face, upper and lower extremities after 2 years of PRP treatment with topical emollients and steroids. Clinical examination and immunological findings were consistent with SSc. Co-existence of these two rare conditions is documented for the first time.

## Introduction

Pityriasis rubra pilaris (PRP) is a rare, chronic erythematous squamous disorder of unknown etiology. It is characterized by follicular plugging, perifollicular erythema, palmoplantar hyperkeratosis and, occasionally, erythroderma [1]. PRP has been found in association with several autoimmune diseases, including thyroiditis, myositis, myasthenia gravis and vitiligo.

Systemic Sclerosis (SSc) is an autoimmune disease characterized by fibrosis of the skin and/or internal organs, small vessel vasculopathy and specific auto antibodies. Herein we report a case of systemic sclerosis in a patient with classic adult pityriasis rubra pilaris.

## Patient and case report

A 38 years-old woman presented in June 2009 with a one year history of progressive widespread symmetrical cutaneous thickening of the skin of the proximal upper extremities, trunk and face, arthralgias, dyspnea on exertion and 10 kg weight loss over the previous 12 months.

**Figure 1:**
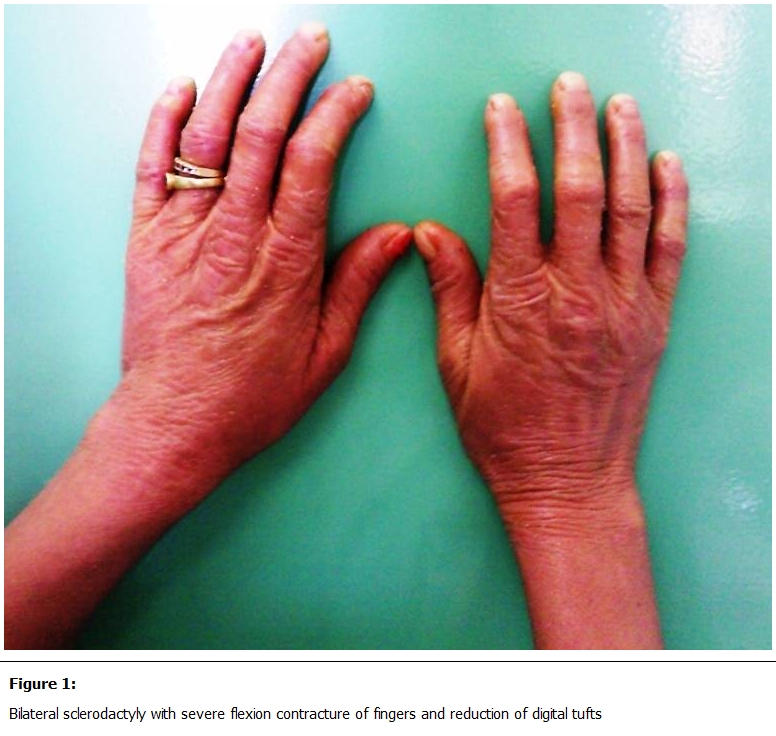
Bilateral sclerodactyly with severe flexion contracture of fingers and reduction of digital tufts

**Figure 2:**
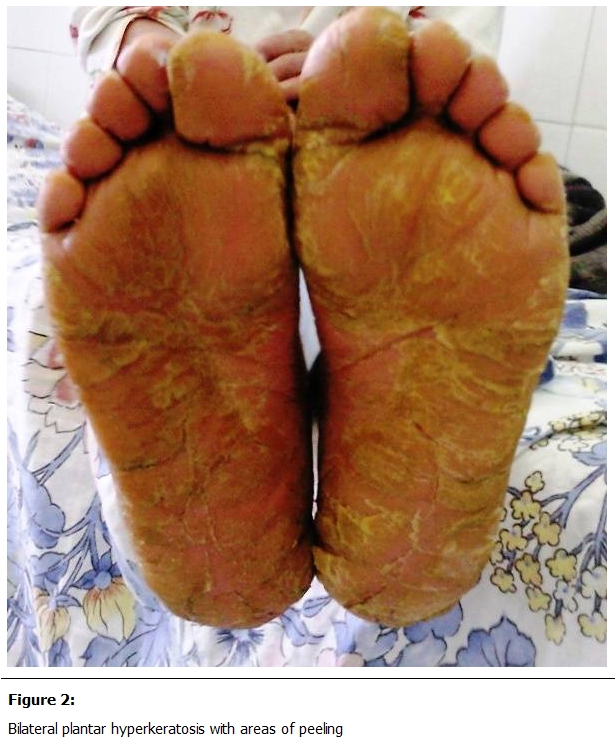
Bilateral plantar hyperkeratosis with areas of peeling

She had been diagnosed with type I adult-onset pityriasis rubra pilaris (PRP) at age 36 years, and had been treated with topical corticosteroids, emollients and cetirizine dichlorhydrate. Family history was negative for skin diseases. Raynaud’s phenomenon was denied. Physical examination revealed a diffuse erythematous desquamative cutaneous eruption with diffuse skin thickening, telangiectasias ans sclerodactyly with finger flexion contractures and digital tuft loss (Figure 1). The palmoplantar surfaces were hyperkeratotic and fissured with areas of peeling (Figure 2). Mouth excursion was limited. Capillaroscopy showed avascular areas and capillary dilatations.

Laboratory tests showed the following results: the erythrocyte sedimentation rate (ESR) 12 mm/h, normal haemoglobin, white blood cells count (WBC) and platelet count., SGOT 32 IU/l, SGPT 44 IU/l. Renal function was normal. The serum calcium, phosphate, protein and creatine kinase level were within the normal limits. Antinuclear antibodies (ANA) were present at 1/1280, in a nucleolar pattern; anti PM-Scl positive.

Histological evaluation of a lesional skin biopsy revealed orthokeratosis and confluent granular layer in the epidermis, and a perivascular lymphohistiocytic cell infiltrate in the papillary dermis consistent with PRP.

Chest radiograph, echocardiogram and electrogram were within normal limits but pulmonary function revealed moderate restrictive disease. Hand radiographs revealed resorption of the distal tufts of several fingers, but no calcifications in the soft tissues. The diagnosis of diffuse cutaneous systemic sclerosis (dcSSc) based on the revised criteria of LeRoy and Medsger [2], associated to classic adult PRP was made. The modified Rodnan skin thickness score was 26. A diligent search for underlying malignant disease was negative, and screening tests for hepatitis B, C and HIV were negative. Oral therapy with colchicine (1 mg per day) was instituted with partial improvement of skin manifestations.

## Discussion

The diagnosis of diffuse cutaneous systemic sclerosis (dcSSc) was based on the revised criteria of LeRoy and Medsger [2] and of pityriasis rubra pilaris, as described above [1].This appears to be the first published description of such an association.

PRP was first described in 1835 by Claudis Tarral [3]. Griffiths proposed classification [1] divides PRP into 5 types on the basis of age of onset, clinical appearance, course and prognosis. A sixth group associated with human immunodeficiency virus (HIV) infection has been suggested [4]. Classic adult type I PRP accounts for over half the occurrences and has the best prognosis. Spontaneous resolution occurs in 80% of patients within 1-3 years [1, 5]. It is characterized by follicular hyperkeratotic papules that coalesce into large, scaly, erythematous plaques, palmoplantar keratoderma, diffuse scaling of the scalp sometimes progressing into erythroderma [1, 6-8]. The affected skin is extremely rough to touch [3]. The histologic features in PRP, although are non-specific, can include irregular acanthosis, alternating orthokeratosis and parakeratosis, hypergranulosis and sparse to moderate lymphocytic perivascular infiltrate of the dermis [7].

The etiology of PRP is unknown. Both familial and acquired forms of the disease have been described [7,9,10]. PRP has been reported in association with HIV infection [11-13], malignancy [4] and Down’s syndrome [14]. PRP has been reported to occur simultaneously with several autoimmune disorders [7,6,15,16] including thyroiditis [15,16], myositis [17], myasthenia gravis [18], coeliac sprue [19] and vitiligo [14]. Seronegative polyarthritis has also been reported in association with PR[17,20-23].

Pityriasis rubra pilaris is confidently diagnosed in this case, based on the age of onset, clinical features, palmoplantar keratoderma and histological findings. Systemic sclerosis is diagnosed on the basis of skin thickening, sclerodactyly, acroosteolysis and presence of anti-nucleolar antibodies, although there was no evidence of objective RP. In fact, SSc is often associated with a short duration or absence of RP before other disease manifestations, early and extensive skin involvement, and earlier occurrence of severe visceral involvement [24].

SS is a rare connective tissue disease of unknown etiology, although there have been several cases report related to environmental agents (various organic solvents, vinyl chloride, silica) or to oil contamination [25]. That latter were taken internally and it is unlikely that the emollients played any role in this case. Scleroderma is thought to arise from a complex and as yet undefined interaction between genetic, environmental and immunologic factors. The fundamental pathogenic process of tissue fibrosis involves interplay between endothelial cell dysfunction and injury, inflammation, auto-immunity and fibroblast activation [26].

Recent reports have suggested that immune dysregulation may also play a role in the pathogenesis of PR [7], based on T-lymphocyte abnormalities [27], lymphocyte hypersensitivity to super antigens [28] and relationship to HIV infection [11-13,29]. It was speculated that the disorder might be the result of an abnormal immune response to some antigenic stimuli [6,8]. Determining whether co-occurrence of PRP and SSc represents a relationship or simply a chance occurrence awaits systematic investigation of each of these rare disorders for evidence of the other disorder recognized here.

The treatment of PRP is often difficult and it has been a source of great interest. Many therapeutic modalities have been employed in its treatment such as Vitamin A and its derivatives [30], methotrexate and cyclosporine A have been used with equivocal outcomes. The use of systemic retinoids has been widely acclaimed. Treatment in our patient relies on topical therapy with emollients and topical steroids with partial improvement of skin lesion. Systemic therapy was restricted to antihistamines. Colchicine was administrated as a first line therapy for its SSc. Finally, the follow-up of our patient should involve regular assessment of cutaneous extension of the two diseases and should be focused on the search of severe visceral involvement of SSc such as pulmonary fibrosis, pulmonary hypertension and renal crises.

## Conclusion

In conclusion, is there an association between PRP and SSc? Our case illustrates for the first time this possible association or co-existence, but the rare nature of the two diseases means a true association is really difficult to prove. PRP has not been previously reported in patients with SSc, and the increase in case reports with this unusual association may lead to explain the relationship between PRP and scleroderma.

## Competing interests

The authors declare no competing interests.
